# Reduced Electron Temperature in Silicon Multi-Quantum-Dot Single-Electron Tunneling Devices

**DOI:** 10.3390/nano12040603

**Published:** 2022-02-11

**Authors:** Youngmin Lee, So Hyun Lee, Hyo Seok Son, Sejoon Lee

**Affiliations:** 1 Quantum-Functional Semiconductor Research Center, Dongguk University-Seoul, Seoul 04620, Korea; ymlee@dongguk.edu; 2 Department of Semiconductor Science, Dongguk University-Seoul, Seoul 04620, Korea; thgus1731@naver.com (S.H.L.); shs_0213@naver.com (H.S.S.)

**Keywords:** quantum dot, single-electron transistor, Coulomb blockade, charge stability, effective electron temperature

## Abstract

The high-performance room-temperature-operating Si single-electron transistors (SETs) were devised in the form of the multiple quantum-dot (MQD) multiple tunnel junction (MTJ) system. The key device architecture of the Si MQD MTJ system was self-formed along the volumetrically undulated [110] Si nanowire that was fabricated by isotropic wet etching and subsequent oxidation of the e-beam-lithographically patterned [110] Si nanowire. The strong subband modulation in the volumetrically undulated [110] Si nanowire could create both the large quantum level spacings and the high tunnel barriers in the Si MQD MTJ system. Such a device scheme can not only decrease the cotunneling effect, but also reduce the effective electron temperature. These eventually led to the energetic stability for both the Coulomb blockade and the negative differential conductance characteristics at room temperature. The results suggest that the present device scheme (i.e., [110] Si MQD MTJ) holds great promise for the room-temperature demonstration of the high-performance Si SETs.

## 1. Introduction

The semiconductor single-electron transistors (SETs), which comprise either the double-barrier tunnel junction (DTJ) with a single quantum dot (QD) or the multiple tunnel junction (MTJ) with multiple quantum dots (MQDs), allow single-electron transport through the discrete quantum energy states of the semiconductor QDs [1,2,3,4,5]. In short, the electron can transfer one-by-one through the quantum states via the Coulomb blockade effect with its corresponding quantum–mechanical single-electron tunneling events. This leads to the unique transfer and output characteristics, such as Coulomb blockade oscillation (CBO) and negative differential conductance (NDC), respectively [6,7,8,9,10,11,12,13,14,15]. For example, the precise control of the single-electron (or even single-spin) transport characteristics was demonstrated on various types of semiconductor QD-based DTJ and MTJ device schemes (e.g., room temperature observation of multiple CBO peaks from multiple quantum states in a Si single-QD device [10], simultaneous observation of both sharp CBO and NDC peaks from a Si-QD DTJ device [9,10,11,12,13,14], bias voltage-controlled precise modulation of energetic Coulomb blockade conditions in a Si single-QD transistor [8], high-fidelity *q*-bit processing in Si MQD [16,17,18,19] and GaAs MQD [20,21,22] devices). Such an extremely high precision of the single-charge manipulation could enable us to extend the SET application toward the broad area of the sensing metrology. Namely, when the external stimuli transfer from the sensing object to the SET, it would significantly influence the electronic charging energy of the QD; hence, the tunneling conductance of the SET could be tuned via changing in the energetic Coulomb blockade condition by the stimuli from the sensing target. In this circumstance, the SET-based sensors could also reveal the higher sensitivity than that of complementary metal–oxide–semiconductor (CMOS)-based sensors, because in the SET, the conductance values at the CBO peaks and valleys (i.e., on- and off-tunneling states) are also precisely controllable by changing the gate and/or the drain bias voltages [23]. Owing to such an astonishing physical mechanism, the electric current standard device [24], the thermometers [25,26], the charge sensors [16,17,18,19], the photon detectors [27,28], the ion sensors [29,30], and the mechanical displacement detectors [1,2,3] were conceived and reported as feasible applications of the SET-based sensors.

In typical SETs, however, the thermal fluctuation and the thermally activated carrier conduction may cause the malfunction of the sensors because those give rise to both the thermal quenching and thermal broadening of CBO and NDC. At the elevated temperature, therefore, the SET will eventually result in the ambiguous operation of the sensors. According to previous literature, thermal quenching and thermal broadening of CBO and NDC are closely relevant to the cotunneling phenomena at the Coulomb blockade state [31,32]. Cotunneling events can be categorized into two different types, i.e., one is elastic cotunneling that occurs via additional electron tunneling through the intermediate virtual quantum levels in the QD, and the other is inelastic cotunneling that takes place via the in- and out-tunneling of other electrons through other quantum levels [33]. These may in turn increase the leakage current at the Coulomb blockade state (i.e., valley current of CBO); hence, the SET will lead to the impairable operation of the SET-based sensors. To increase the efficiency of the SET-based sensors, therefore, suppressing the cotunneling effect is vital. Furthermore, since electron cotunneling strongly relies on the effective electron-temperature in the QD device, the low operation temperature of the conventional SETs makes the thermal fluctuation and the leakage current issues more critical.

All these above backgrounds prompt us to investigate the fabrication and the characterization of the high-performance room-temperature-operating SETs, in which the cotunneling behaviors should be effectively suppressed. Herein, to take full advantage of the scientific and technical knowledge on the Si nanoelectronic devices, we fabricated and characterized the CMOS-compatible Si SETs that could steadily operate at room temperature. The devices were devised in the form of the gate-all-around (GAA) Si nanowire-channel metal–oxide–semiconductor field-effect transistor (MOSFET), where the MTJs were self-formed through isotropic wet etching of the undulated [110] Si nanowire that had been created by *e*-beam lithography. The transport characteristics of the fabricated SETs are thoroughly examined, and their effective electron temperatures are analyzed and discussed by means of the cotunneling current characterization.

## 2. Experimental Section

**Figure 1:**
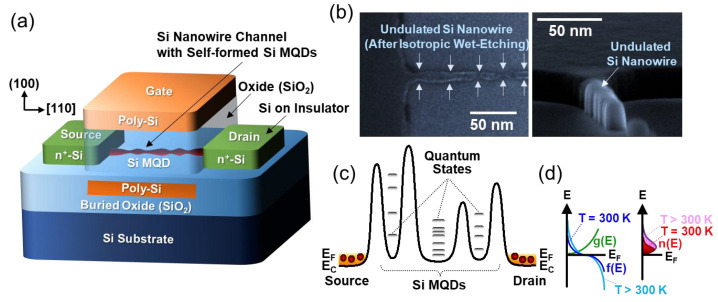
(**a**) Schematic of the fabricated SHT device, (**b**) scanning electron microscopy images (left, top view; right, tilted view) of the undulated Si nanowire channel obtained from dummy samples, (**c**) expected energy band diagram of the undulated Si nanowire-channel at the conduction band region, and (**d**) Fermi–Dirac distribution function, f(E) at T = 300 K and T > 300 K; density of state function, g(E); and electron distribution function, n(E) at T = 300 K and T > 300 K in the source and the drain reservoirs. In (**c**,**d**), E_c_ and E_F_ denote the conduction band and the Fermi level, respectively.

Figure 1a displays the schematic illustration of the Si SET, which comprises a device scheme of the CMOS-compatible Si nanowire-channel GAA MOSFET. To construct such a device architecture, as a primary task, the [110] Si nanowire-channel (length ≈ 200 nm, width ≈ 40 nm) was patterned on the ~10 nm-thick silicon-on-insulator substrate by using *e*-beam lithography. Next, to shrink the volumetric nanowire size, isotropic wet etching was carried out by using the SC-1 solution (NH_4_OH:H_2_O_2_:H_2_O = 1:1:6). Then, the size of the etched nanowire (≈15 nm) became narrower than the initial size (≈40 nm) of the *e*-beam-lithographically patterned Si nanowire (Figure 1b). To configure the GAA structure, subsequently, a part of the buried oxide underneath the Si nanowire-channel was etched out by dipping the sample into the dilute hydrogen fluoride acid solution (HF:H_2_O = 1:10). Then, the Si nanowire-channel could be suspended from the buried oxide because of the large supporting areas of source (S) and drain (D). Thereafter, the surface of the suspended Si nanowire was oxidized by dry oxidation at 900 °C to form the gate oxide layer. During this step, the final diameter size of the Si nanowire was further shrunken down to <5 nm [7,8,9,10,11]. Through the sequential deposition of additional SiO_2_ (≈30 nm) and *n*^+^ poly-Si (≈250 nm) gate (G), finally, the formation of the GAA stacks was finalized. The rest processes for forming the *n*^+^-S (≈10^20^ cm^−3^) and *n*^+^-D (≈10^20^ cm^−3^) reservoirs were followed by the CMOS-compatible process steps with the P^+^ ion implantation and the thermal activation of the dopants at 950 °C.

Here, one needs to remind that, during the volumetric shrinkage of the Si nanowire by isotropic wet etching, the diameter sizes of the nanowire were volumetrically undulated along the direction normal to the channel axis (Figure 1b) and such a volumetric undulation would become significant during the thermal oxidation of the Si nanowire surface. Since the diameter size of the Si nanowire became narrow (≈5 nm), the volumetrically undulated areas would be much narrower than 5 nm. Due to the strong quantum-mechanical sub-band modulation in [110] Si nanowires [34,35], in the volumetrically undulated Si nanowire, the MTJ system could be self-created along the nanowire–channel direction. In short, some parts of the Si nanowire would be squeezed (<<5 nm), and the rest of the parts (≈5 nm) would be connected in series along with the squeezed regions. According to Refs. [36,37], the sub-band modulation becomes significant as the diameter of the [110] Si nanowire decreases. For example, in the [110] Si nanowire with the smaller diameter of <2 nm, the ground state could locate at 500 meV above the conduction band (E_C_) of bulk Si [36,37]. This would eventually create the energy band fluctuation at E_C_ along the Si nanowire (Figure 1c). Accordingly, the squeezed regions (<<5 nm) and the unsqueezed areas (≈5 nm) may act as the tunneling barriers and QDs, respectively. Hence, the MTJ system could be formed along the Si nanowire for the fabricated device to operate as a MQD Si SET. In this circumstance, the single-electron tunneling transport would strongly depend on both the quantum level spacings of the QDs and the carrier distribution functions of the electron reservoirs (Figure 1d). Therefore, the Coulomb blockade characteristics would strongly rely on the effective electron temperature and its corresponding cotunneling effect.

## 3. Results and Discussion

**Figure 2:**
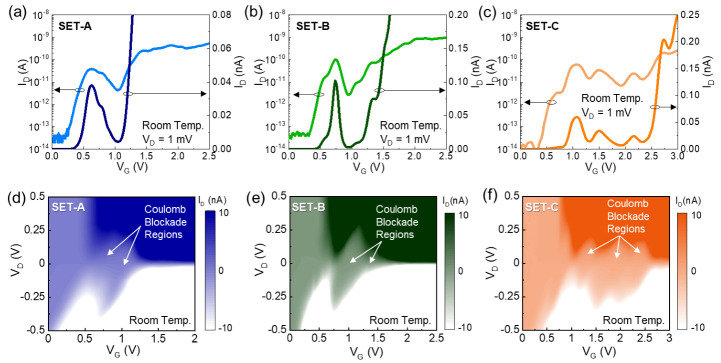
Transfer characteristic curves (i.e., I_D_–V_G_ at V_D_ = 1 mV) at room temperature of (**a**) SET-A, (**b**) SET-B, and (**c**) SET-C; and contour plots of I_D_ as functions of V_G_ and V_D_ at room temperature for (**d**) SET-A, (**e**) SET-B, and (**f**) SET-C.

Figure 2 shows the transfer characteristics of three different SETs that were fabricated through the identical process procedures described above. For convenience only, we simply refer to the three SETs as SET-A, SET-B, and SET-C, respectively. Figure 2a–c displays the drain current vs. gate voltage (I_D_–V_G_) curves of SET-A, SET-B, and SET-C under the drain voltage (V_D_) of 1 mV at room temperature, respectively. The SETs reveal the typical transfer characteristics of the MQD SET. Namely, the devices exhibit the clear CBO peaks together with the multiple humps, arising from the stochastic tunneling events in the MTJ system [38,39]. Since the peak and valley of CBO correspond to the on- and off-resonance states for single-electron tunneling via the Coulomb blockade event, the large magnitude of the maximum peak-to-valley current ratio (>20) depicts the large quantum level spacings to exist in the QDs. As aforementioned, the QDs were self-formed along the volumetrically undulated Si nanowire-channels. In such a geometrical structure, the large sub-band modulation at the squeezed nanowire regions (<<5 nm) could create the large potential barriers, which are big enough to energetically separate the unsqueezed nanowire areas (≈5 nm). According to Refs. [36,37], as the diameter of the [110] Si nanowire decreases, the quantum level spacings could increase up to 75 meV because of the decreased effective mass (*m*_e_ ~ 0.11*m*_0_ [36]) at the two-fold Γ valley. One can therefore conjecture the unsqueezed nanowire areas to act as the QDs, possessing the large quantum level spacings. Accordingly, the fabricated devices could operate as room-temperature-operating MQD SETs.

The MQD behavior of the fabricated SETs can also be traced from the charge stability diagram (i.e., Coulomb diagram). Figure 2d–f shows the contour plots of I_D_ as functions of V_G_ and V_D_ for SET-A, SET-B, and SET-C, respectively. The SETs clearly display the typical shape of the rhombus Coulomb blockade regions, indicative of the single-electron tunneling transport characteristics via the Coulomb blockade effect. Here, it should be noted that some parts of the Coulomb blockade regions are overlapped by their adjacent Coulomb blockade regions. Such an overlapped blockade feature can be interpreted by the irregular MQD system. As can be expected from Figure 1a–c, both the tunnel barrier heights and the quantum-dot sizes would be inhomogeneous in the present devices because those were self-created through the volumetric shrinkage of the undulated Si nanowire. In this case, the shapes and sizes of the QDs as well as the heights and curvatures of the tunnel barriers would be irregular so that the QDs would have different quantum level spacings. Since such an inhomogeneity causes the imbalance of the energetic Coulomb blockade conditions for every dot in MQDs, the present type of the MQD system would show the stochastic tunneling characteristics, resulting in the appearance of the overlapped Coulomb blockade regions in the Coulomb diagram.

Here, we also note that the three SETs reveal quite different Coulomb blockade features, even though those were fabricated in a same bath with the identical fabrication process. As mentioned earlier, the energy band profile of the present device scheme strongly depends on the degree of volumetric undulation along the nanowire channel direction. In this case, the energy band profile would alter device by device because the strong and weak sub-band modulations at the squeezed and unsqueezed areas are responsible for the self-formation of both the tunnel barriers and the isolated dots. In brief, the inhomogeneity of volumetric undulation leads to the randomness of the MQD MTJ profile with the different number of QDs. Accordingly, the SETs fabricated in a single chip showed different CBO features (Figure S1, Supplementary Materials). In the application point of view, such inhomogeneous device characteristics may restrict the reliability of the circuit integration. Hence, the key issue could become the fabrication of the device structure with a clear and regular succession of quantum dots at regular distances. To release this issue, therefore, the advanced sub-5 nm patterning techniques can be suggested as feasible ways to improve the device homogeneity. For example, recent advances in nanofabrication technology, such as scanning probe lithography [40], heavy ion lithography [41], extreme ultraviolet lithography [42,43], block copolymer self-assembly [44], may allow the precise undulation of the Si nanowire because these methods enable us to control both the fine size and the exact site of the sub-5 nm patterns.

In the MQD system, the cotunneling effect can be effectively suppressed because of the following reason. According to the single-electron tunneling transport model [32,33], the magnitude of I_D_ is proportional to the multiplication factor;

(1) $${\left(g_{T}e^{2}/h\right)}^{N+1}$$

where $g_{T}$ is the tunnel conductance of the single tunnel barrier, *e* is the unit charge, *h* is the Planck constant, and *N* is the number of QDs. Since the magnitude of ($g_{T}e^{2}/h$) is much smaller than 1 at the Coulomb blockade state (i.e., very low $g_{T}$ at the off-tunneling state), the multiplication factor ${\left(g_{T}e^{2}/h\right)}^{N+1}$ would drastically decrease with increasing *N* in the MQD system. To briefly sum up, the cotunneling current (i.e., valley current (I_valley_)) could be effectively decreased as one increases the number of QDs. Based upon the above model, for the MQD system with *N* QDs, the value of I_valley_ can be described by [45]

$$I_{\mathrm{valley}}\propto {\left(g_{T}e^{2}/h\right)}^{N+1}{\left\{{\left(eV_{D}\right)}^{2}+{\left(2\pi k_{B}T_{\mathrm{eff}}\right)}^{2}\right\}}^{N}V_{D}$$

(2) $$\equiv G_{b}^{N+1}{\left\{{\left(eV_{D}\right)}^{2}+{\left(2\pi k_{B}T_{\mathrm{eff}}\right)}^{2}\right\}}^{N}V_{D}$$

where $G_{b}^{N+1}$ is the multiplication of the tunnel barrier conductance, *k_B_* is the Boltzmann constant, and *T*_eff_ is the effective electron temperature. For example, the I_valley_ values for the single (*N* = 1), double (*N* = 2), and triple (*N* = 3) QD systems can be derived by Equations (3)–(5), respectively [9,10]:

(3) $$I_{\mathrm{valley}\left(N=1\right)}=\alpha G_{S}G_{D}\left\{e^{2}V_{D}^{3}+{\left(2\pi k_{B}T_{\mathrm{eff}}\right)}^{2}V_{D}\right\}$$

(4) $$I_{\mathrm{valley}\left(N=2\right)}=\beta G_{S}G_{i}G_{D}\left\{e^{4}V_{D}^{5}+2e^{2}{\left(2\pi k_{B}T_{\mathrm{eff}}\right)}^{2}V_{D}^{3}+{\left(2\pi k_{B}T_{\mathrm{eff}}\right)}^{4}V_{D}\right\}$$

(5) $$I_{\mathrm{valley}\left(N=3\right)}=\gamma G_{S}G_{i1}G_{i2}G_{D}\left\{e^{6}V_{D}^{7}+3e^{4}{\left(2\pi k_{B}T_{\mathrm{eff}}\right)}^{2}V_{D}^{5}+3e^{2}{\left(2\pi k_{B}T_{\mathrm{eff}}\right)}^{4}V_{D}^{3}+{\left(2\pi k_{B}T_{\mathrm{eff}}\right)}^{6}V_{D}\right\}$$

where *α*, *β*, and *γ* are the proportional factors, and *G_S_*, *G_i_*, and *G_D_* are the source, intermediate, and drain conductance values, respectively.

**Figure 3:**
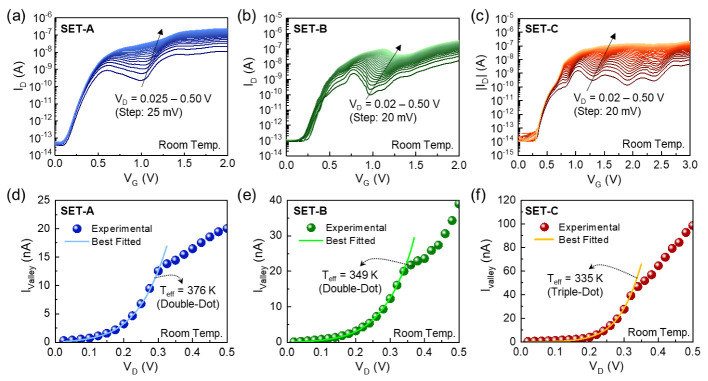
Evolution of the CBO peaks at the positive V_D_ region (i.e., I_D_–V_G_ curves at V_D_ = 0.02 – 0.5 V) for (**a**) SET-A, (**b**) SET-B, and (**c**) SET-C; and the I_valley_ as a function of V_D_ for (**d**) SET-A, (**e**) SET-B, and (**f**) SET-C.

To assess the cotunneling characteristics of the present devices, we examined the V_D_ dependence of the CBO evolution (Figure 3a–c) and plotted the values of I_valley_ as a function of V_D_ (Figure 3d–f). As can be seen from Figure 3a–c, the devices exhibit the clear valley states even at higher V_D_ up to 0.5 V. In general, the cotunneling events would become significant at the higher bias voltages because the higher external electric field from the over-driving voltage gives rise to the increase in the excess energy in the QD system [15,46,47]. Therefore, the clear valley states at higher V_D_ depict the present devices to hold a weak cotunneling effect. Nevertheless, the magnitude of I_valley_ goes out of the single tendency when V_D_ exceeds 0.3–0.35 V. In the present type of the SETs, the tunnel barriers are created by the sub-band modulation at the squeezed Si nanowire regions but not the material barriers, such as SiO_2_. In this case, the tunnel barriers would be lowered with increasing V_D_, particularly at the drain region, because the tunnel barrier is capacitively coupled in between the dot and the electrode. Thus, the stochastic tunneling events would alter and/or be broken at the higher V_D_ region so that the I_valley_ values become irrespective of the above cotunneling model. For data fitting to the above equations, we therefore chose only the V_D_ region, in which I_D_ follows the I_valley_ vs. V_D_ functions in Equations (3)–(5).

By fitting the measured I_valley_ values to the above equations, we found that the SET-A, SET-B, and SET-C devices were composed of the MQD systems with N = 2 (double), 2 (double), and 3 (triple), respectively. Namely, the I_valley_ data could be well fitted only to Equation (4) for SET-A and SET-B and to Equation (5) for SET-C. From the fitting curves, the *T*_eff_ values were determined to be 376, 349, and 335 K for SET-A, SET-B, and SET-C, respectively. Accordingly, the excess energy (*E*_exc_ = *E*_e–f_ − *E*_env_, where *E*_env_ is the thermal energy at the environmental system) could be deduced to be 6.5, 4.2, and 3.0 meV for SET-A, SET-B, and SET-C, respectively. In addition, the other SETs (*N* = 2 or 3) fabricated in a single chip were confirmed to have similar values to the above (Figure S1, Supplementary Materials). These values are much smaller than those of other single-dot SETs/SHTs and are comparable to those of the state-of-the-art single-dot SETs that comprised the ellipsoidal QDs produced by sophisticate fabrication processes (Table 1). As a result, forming the MQD system would effectively lead to the decrease in the cotunneling effect; hence, the Coulomb blockade state (i.e., valley state) could be stabilized even at higher V_D_.

**Figure 4:**
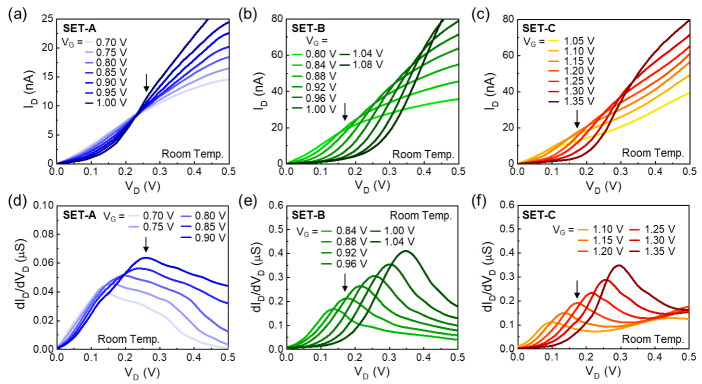
I_D_–V_D_ output characteristic curves at various V_G_ near the Coulomb blockade regions for (**a**) SET-A, (**b**) SET-B, and (**c**) SET-C, and dI_D_/dV_D_–V_D_ curves of (**d**) SIA, (**e**) SET-B, and (**f**) SET-C.

As mentioned earlier, *T*_eff_ affects not only the cotunneling characteristics at the Coulomb blockade states, but also the thermally activated carrier conduction (i.e., thermal fluctuation of the quantum states). To verify the energetic stability of the quantum states, we examined the NDC characteristics via observing the V_D_-dependent single-electron tunneling current at V_G_ near the Coulomb blockade state. Figure 4a–c displays the room-temperature I_D_–V_D_ characteristic curves at various V_G_ conditions near the Coulomb blockade regions for SET-A, SET-B, and SET-C, respectively. All the devices clearly exhibit the I_D_ humps or knees, as indicated by the arrows. For example, in the case of SET-A (Figure 4a), the I_D_ hump begins to appear at V_G_ = 0.7 V, and the position of the I_D_ hump gradually moves to the higher V_D_ and higher I_D_ region as V_G_ increases.

A similar feature can be also observable from SET-B (Figure 4b) and SET-C (Figure 4c). Namely, SET-B and SET-C show the I_D_ knees in their I_D_–V_D_ characteristic curves. As shown in Figure 4d–f, the I_D_ humps and knees can be confirmed to originate from the NDC characteristics. These are attributable to the sudden drop of the drain conductance due to the off resonance at the forbidden energy gaps [10,12]. In other words, the tunneling processes could be prohibited at specific V_D_ bias voltages because of the large quantum level spacings in the ultra-small Si QDs. Based upon all the above results, therefore, it can be concluded that both the cotunneling effects and the thermal fluctuation behaviors could be effectively reduced by forming the MQD system. Furthermore, the Si MQD system formed along the [110] Si nanowire can be suggested as a commendable strategy to reduce the *T*_eff_ value for the room-temperature application of the CMOS-compatible Si SETs.

## 4. Summary and Conclusions

The CMOS-compatible Si MQD SETs were fabricated in the form of the Si nanowire-channel MOSFETs, in which the multiple Si QDs were self-formed through isotropic wet etching of the *e*-beam-lithographically patterned [110] Si nanowires. Owing to the large sub-band modulation in the volumetrically undulated [110] Si nanowire, the Si MQD MTJ system with large quantum level spacings could be achieved. Although the volumetrically undulation method (i.e., self-formation of the Si MQD MTJ system) did not fully guarantee the identical Coulomb blockade characteristics for all the devices in a single chip, in terms of the theoretical fitting model, we found that the MQD MTJ system could allow us to effectively reduce both the cotunneling current and the effective electron temperature. These eventually led to the room-temperature manipulation of clear CBO and NDC peaks at wide bias voltage ranges. Consequently, the formation of the [110] Si MQD MTJ system could be an effective strategy to fabricate the high-performance CMOS-compatible Si SETs.

## Figures and Tables

**Table 1:** Comparison of *T*_eff_ and *E*_exc_ for various CMOS-compatible Si SETs with different device configurations.

Number of QD	Device Type	Si Nanowire Direction	*T*_eff_ (K)	*E*_exc_ (meV)	Ref.
Single	Si SHT	[100]	1260	82.7	[15]
	Si SHT	[100]	870	49.1	[15]
	Si SHT	[100]	415	9.9	[15]
	Si SET	[110]	312	1.0	[10]
	Si SET	[100]	338	3.3	[9]
Double	Si SET (A)	[110]	376	6.5	This Work
	Si SET (B)	[110]	349	4.2	
	Si SET (S1)	[110]	384	7.2	
	Si SET (S2)	[110]	389	7.7	
	Si SET (S3)	[110]	397	8.4	
Triple	Si SET (C)	[110]	335	3.0	
	Si SET (S4)	[110]	342	3.4	

## Data Availability

Not applicable.

## Supplementary Materials

The following supporting information can be downloaded at: https://www.mdpi.com/article/10.3390/nano12040603/s1, Figure S1: Coulomb blockade characteristics of (a) SET-S1, (b) SET-S2, (c) SET-S3, and (d) SET-S4 that had been fabricated in a single chip studied in the present work.
