# Supplementary material for: Reduced Electron Temperature in Silicon Multi-Quantum-Dot Single-Electron Tunneling Devices
Source: Nanomaterials (Basel). 2022 Feb 11;12(4):603. doi: 10.3390/nano12040603 (PMC8876062; doi:10.3390/nano12040603)
Supplement: Supplementary file 1 [file nanomaterials-12-00603-s001.zip › nanomaterials-1566975-supplementary.pdf]

nanomaterials

MDPI

Supplementary Materials

Reduced Electron Temperature in Silicon Multi-Quantum-Dot Single-Electron Tunneling Devices

Youngmin Lee [1], So Hyun Lee [2], Hyo Seok Son [2] and Sejoon Lee [1,2,*]

[1] Quantum-functional Semiconductor Research Center, Dongguk University-Seoul, Seoul 04620, Korea; ymlee@dongguk.edu

[2] Department of Semiconductor Science, Dongguk University-Seoul, Seoul 04620, Korea; thgus1731@naver.com (S.H.L.); shs_0213@naver.com (H.S.S.)

* Correspondence: sejoon@dongguk.edu

Coulomb Blockade Characteristics of Other SETs Fabricated in a Single Chip

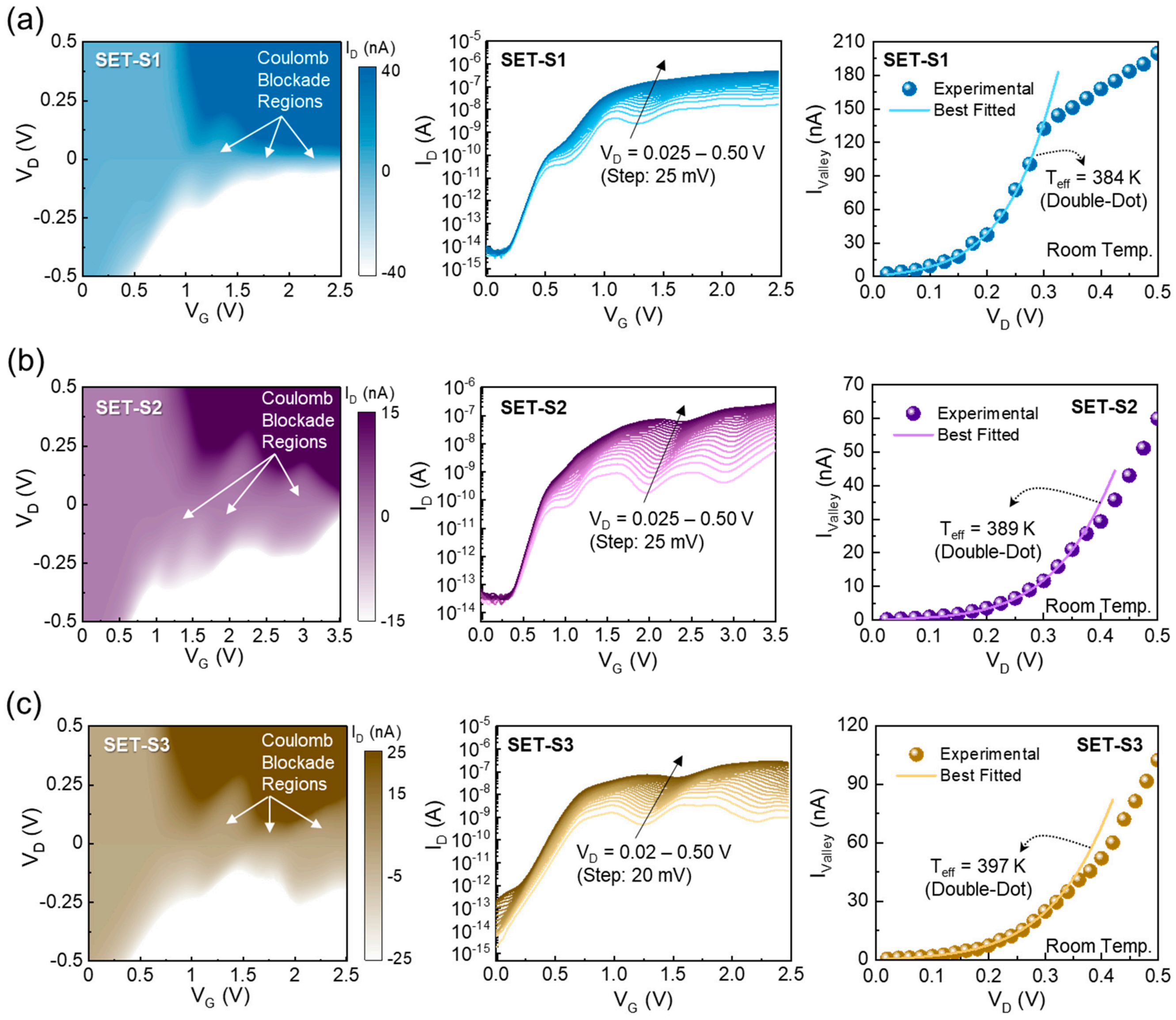

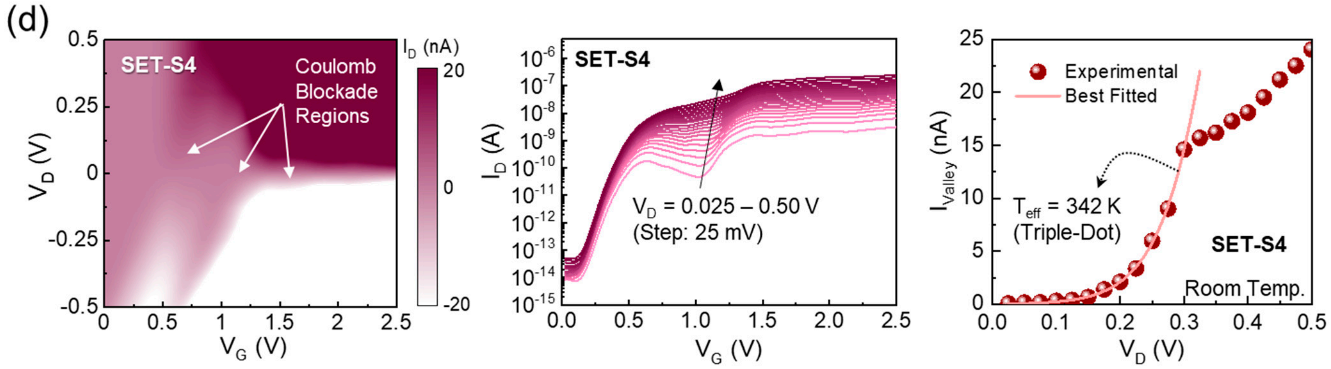

Figure S1. Coulomb blockade characteristics of (**a**) SET-S1, (**b**) SET-S2, (**c**) SET-S3, and (**d**) SET-S4 that were fabricated in a single chip studied in the present work.
